# Genetic transformation and fluorescent protein expression in the biomaterial-producing fungus *Fomes fomentarius*

**DOI:** 10.1186/s40694-026-00228-w

**Published:** 2026-11-02

**Authors:** Carmen Regner, Justus Zillessen, Friederike Hoberg, Svenja Elsner, Timothy C. Cairns, Carsten Freidank-Pohl, Vera Meyer

**Affiliations:** Department of Applied and Molecular Microbiology, Technische Universität Berlin, Strasse des 17. Juni 135, 10623 Berlin, Germany

**Keywords:** Mycelium, Material, Genetic transformation, Tinder fungus, *Fomes fomentarius*

## Abstract

**Background:**

Fungal-based materials are currently studied as renewable and sustainable alternatives to fossil-based materials used in construction, packaging, textile, and fashion industries. The polypore fungus *Fomes fomentarius* produces a range of lightweight, durable, and flexible materials from diverse plant residual streams. Genetic engineering of this fungus is an important technological objective, both from fundamental perspectives and to optimize performance characteristics of *F. fomentarius* materials.

**Results:**

We provide proof of principle that *F. fomentarius* can be genetically transformed. Hyphae produced high amounts of protoplasts using standard protocols. These protoplasts were transformed using nourseothricin selection and a nourseothricin N-acetyl transferase resistance marker (*natR*). Expression of *natR* was successful using a *F. fomentarius* translation elongation factor promoter. Stable, multicopy, and non-targeted integration of exogenous cassettes with recipient genome was confirmed by diagnostic PCR and Southern blotting. Preliminary attempts at genome editing based disruption of a putative *ku70* encoding gene were, however, unsuccessful. We validated expression of two fluorescent proteins using a *Schizophyllum commune* H2B promoter using qRT-PCR and fluorescent microscopy. These data confirm functionality of mCherry and eGFP in *F. fomentarius* and suggest these proteins can be used for future protein localisation studies. Finally, we report DNase activity in *F. fomentarius* which we hypothesize may be a significant technical challenge for genetic transformation of this fungus.

**Conclusion:**

*F. fomentarius* can be genetically transformed using the promoters, marker, and nourseothricin selection system developed in this study. Expression of both eGFP and mCherry is a significant step towards cell biological and gene functional analyses using fluorescent microscopy. We have laid the foundations for overexpression studies, and, additionally, identified several challenges which need to be overcome for targeted genetic engineering in this important material producing fungus.

**Supplementary Information:**

The online version contains supplementary material available at https://doi.org/10.1186/s40694-026-00228-w.

## Introduction

Woody plants cover over 30% of the earths land area [1]. Trees, shrubs, and certain vine species use wood- which is a complex mixture of cellulose, hemicellulose, and lignin polymers- for both structural support and water transport. Wood is extremely difficult to degrade, both in terms of enzymatic breakdown and physical penetration of the substrate. Basidiomycete fungi are of fundamental importance for nutrient recycling of woody plants in natural ecosystems, managed forests, and agriculture [2]. Brown- and white-rot fungi are able to degrade cellulose and hemicellulose, either enzymatically or via reactive oxygen species and radicals. White-rot fungi, however, are the only known organisms to efficiently and completely degrade lignin [3]. With regards to physical penetration of wood, the remarkable turgor pressure of filamentous hyphae enables fungal colonisation deep into the substrate [4]. Nutrient recycling of woody plants by basidiomycetes can therefore be viewed as multiple interconnected and dynamic processes, including sensing, secretion, enzymatic hydrolysis, penetration, mycelial colonization, and physical binding of fungal cells to the plant .

Over the last 20 years, material scientists and biotechnologists have been repurposing wood-degrading fungi to generate a diverse range of sustainable biomaterials [5–7]. Here, plant material is colonized by fungal hyphae to generate composite or non-composite materials with a range of desired parameters, such as size, shape, weight, or tensile strength. Fungal-based materials are currently prospected as alternatives to established textiles, packaging, and building materials in many industries, including fashion, automotive, electrical, and construction [5–7]. Nearly 30 different filamentous fungal species have been experimentally confirmed as suitable for material production, most of which belong to the white-rot basidiomycetes including *Ganoderma lucidum*, *Schizophyllum commune*, *Pleurotus ostreatus*, *Trametes versicolor*, and *Fomes fomentarius* [5–7]. Performance of composite materials produced with these materials - such as tensile strength, flammability, and weight - is often competitive with conventional materials such as expanded polystyrene [8, 9]. With regards to sustainability, quantitative comparisons between fungal-plant composites and conventional materials - such as concrete- demonstrate biomaterials perform favourably with regards to water usage, air pollution, and the emission of greenhouse gases [9–11]. Given that fungal-based composites effectively sequester carbon in the plant-derived substrate, they constitute an important technology for the transition from a petroleum to bio-based economy [12]. Indeed, the utility and sustainability of fungal-based materials is evidenced by several multinational companies producing their products commercially [7].

Despite the well-established advantages of fungal-based materials, the underlying biology of wood-degrading fungi during composite formation remains largely uncharacterized, especially when compared to medical and plant mycology of basidiomycetes such as *Cryptococcus neoformans* [13] and *Ustilago maydis* [14]. Encouragingly, genetic engineering studies in material producing fungi have in the last decade begun to answer many important questions. This includes, for example, how cell surface hydrophobins impact material formation in *S. commune* [15] and *P. ostreatus* [16]. Additionally, laccase activity and lignin modification have been interrogated in *G. lucidum* [17], and other important species [18]. Genes encoding various regulatory proteins have also been functionally analysed, including transcription factor Ace3 in *Dichomitus squalens* [19], PacC in *G. lucidum* [17], and a wide range of developmental regulators in *S. commune* [20]. From a methodological perspective, these studies have utilized a diverse range of advanced genetic tools, including classical deletion, over-expression, genome editing and RNAi. These genetic engineering technologies will enable fundamental and applied advances in the biomaterials field.

We have recently reported the use of the white-rot agaricomycete *F. fomentarius* as cell factory to generate a diverse range of fungal-based materials including composites, foams, and paper based on moulding and 3D-printing techniques [8, 21–23]. We consider genetic engineering to be a high priority methodological advance for understanding and optimizing materials produced by this fungus, but no transformation has been described to date.

In this study, we provide the first proof that *F. fomentarius* can be genetically transformed. Protoplasts were generated using standard enzymatic removal of the hyphal cell wall and subsequent osmotic buffering. We confirm a nourseothricin N-acetyltransferase encoding cassette is a functional selection marker in this fungus using a native promoter from *F. fomentarius*. Multicopy and stable integrations of exogenous cassettes with the recipient genome were demonstrated via Southern blot. Attempts to target a *ku70* encoding gene using homology flanks and genome editing were not successful. Finally, we express fluorescent proteins for the first time in this fungus, strongly suggesting that eGFP and mCherry are suitable for future subcellular analyses and protein localization studies. Taken together this study constitutes a significant advance for genetic engineering of *F. fomentarius*.

## Methods

### Strains used in this study

The dikaryotic *F. fomentarius* isolate PaPF11 was used throughout this study [8]. *Aspergillus niger* N402 [24] was used for DNase activity assays. Routine cultivation of *F. fomentarius* was performed with malt extract broth (MEB, Carl Roth) or malt extract agar (MEA, Carl Roth), with cultures incubated in a temperature-controlled growth room at 25 °C. Where indicated, complex media (CM), consisting of 1.0% (w/v) glucose, 0.1% (w/v) casamino acids, 0.5% (w/v) yeast extract, 70 mM NaNO₃, 2 mM MgSO₄·7 H₂O, 11 mM KH₂PO₄, 7 mM KCl, and 0.1% (v/v) trace elements was used for growth and supplemented with 1.5% (w/v) agar added for solid medium [25].

### Construction of plasmid pMC_2_90 using modular cloning (MoClo)

All primers used in this study are given in Supplemental Tables 1 and all plasmid maps are given in Supplemental File 1. The genome sequence of *F. fomentarius* CIRM-BRFM 1821, assembly GCA_022606135.1 was used as reference for designing all primers and planning the cloning steps. SnapGene (GSL Biotech, USA) was utilized for the pMC_2_90 *in-silico* workflow including nucleic acid design, restriction cloning, and examination of Sanger sequencing results. PCR amplifications were carried out using KAPA HiFi HotStart ReadyMix (Roche, Switzerland) and Phire Hot Start II DNA-Polymerase (Thermo Fisher Scientific, USA). Templates for the Level 0 assembly were obtained from PaPF11 genomic DNA or plasmid pHom2. All vectors were constructed utilizing the MoClo system [26]. Correct plasmid assembly was identified via blue/white screening and subsequently verified by Sanger sequencing. All MoClo assemblies conducted during this study used an over-night programme for ligation (50 cycles of 5 min 37 °C followed by 4 min 16 °C, a final digest step at 37 °C for 10 min) and enzyme denaturation at 80 °C for 10 min. Per 15 µl reaction, 20 fmol backbone and a fourfold excess of all inserts where ligated with 1 µl of T4 DNA ligase and 1 µl of BpiI or BsaI (all from ThermoFisher Scientific). Level 2 MoClo assembly was employed to surround the ORFs with 250 bp up-/downstream arms homologous to the *ku70* locus in PaPF11. A list of the final vectors created in this study can be found in Table 1. All donor DNA (dDNA) cassettes were obtained via PCR with Q5 polymerase (New England Biolabs GmbH, Germany) and purified using innuPREP DOUBLEpure Kit (IST Innuscreen GmbH, Germany).

### Construction of plasmids pMC_2_115 and pMC_2_116 using Gibson assembly

For generation of plasmids to test expression of fluorescent proteins, pMC_2_115 and pMC_2_116 were constructed by amplifying the expression cassette for H2B-eGFP and H2B-mCherry from plasmid p40 and p14 and integrated into the PCR-amplified plasmid backbone derived from pMC_2_91 using the primers listed in Supplemental Table 1. All PCRs were performed with Kapa HiFi polymerase (Roche) at an annealing temperature of 66 °C. For Gibson cloning, the Gibson Assembly HiFi Master Mix (ThermoFisher) was used according to the manufacturer instructions. Chemically competent *E. coli* Top10 (ThermoFisher Scientific) was used for transformation of the assembly mix and plasmid propagation. Correctness of assembled plasmids was verified by whole-plasmid sequencing (GENEWIZ Germany GmbH).

**Table 1:** Plasmids used or constructed in this study

Name	Genotype	Notes	References
pHom2	*1000 bp-hom2-5’-flank-pScomgpd-natR-tScomSC3-1000 bp-hom2-3’-flank*		[20]
pMC_2_90	*ku70-250 bp-Ffomtef1-natR-tScomSC3-ku70-250 bp*	Based on pAGM4273 backbone	This study
p14	*pScomgpd-BleoR-tScomgpd; pScomH2B-ScomH2B::mCherry-tScomCdc42*	Used for amplification of H2B-mCherry cassette	[27]
p40	*pScomgpd-BleoR-tScomgpd; pScomH2B-ScomH2B::eGFP-tScomCdc42*	Used for amplification of H2B-eGFP cassette	[28]
pMC_2_91	*ku70-250 bp-Ffomtef1-gpd1intron-natR-tScomSC3-ku70-250 bp*	Used for insertion of fluorescent protein cassettes from p14 and p40	This study
pMC_2_115	*Ffomtef1-gpd1intron-natR-tScom*,* pScomH2B-ScomH2B::mCherry-tScomCdc42*		This study
pMC_2_116	*Ffomtef1-gpd1intron-natR-tScom*,* pScomH2B-ScomH2B::eGFP-tScomCdc42*		This study

Maps of plasmids generated in this work are given in Supplemental File 1

### In silico analysis of *tef1* promoter

The native promoter sequences were analysed using the YAPP Eukaryotic Core Promoter Predictor (https://www.bioinformatics.org/yapp/cgi-bin/yapp.cgi) to determine the TSS and locate cis-regulatory elements.

### *F. fomentarius* transformation

The transformation protocol was adapted based on our established methods for *A. niger* transformation [29, 30]. In brief, cryopreserved PaPF11 was plated on CM agar and incubated for seven days at 25 °C. Cultures were homogenized in CM and transferred to Fernbach flasks for static culture incubation, 5–7 days at 25 °C. In brief, the biomass was cut up into pieces and incubated in protoplastating solution (200 mM NaH_2_PO_4_, 800 mM sorbitol, pH 6.0 containing 25 mg/ml VinoTaste^®^ Pro, (Novozymes, Denmark), 10 mg/ml BSA (Carl Roth GmbH & Co. KG, Germany), 0.16 µl/ml Chitinase (Asa Spezial Enzyme GmbH, Germany) at 37 °C and 65 rpm until a sufficient degree of protoplastating was achieved (~ 1.5 h). Formation of protoplasts was verified via light microscopy at 400x magnification (Leica DM5000 CS, Germany). The protoplasts were purified by filtration through Miracloth (Merck Millipore) into 50 ml Falcon tubes (Corning) and pelleted at 4 °C and 1500×g^− 1^ for 10 min using a swing-out rotor centrifuge. After two subsequent washing steps with STC (800 mM sorbitol, 50 mM CaCl_2_, 10 mM Tris, pH 7.5), protoplast concentration was determined using a hemocytometer (Thoma chamber). Approximately 6 × 10^7^ protoplasts were used per transformation reaction. Cas12a-RNP complexes were prepared at room temperature (RT) prior to the transformation. The transformation reactions were in 15 ml Falcon tubes by adding the donor DNA to 33 µl of 60% PEG 4000 (Carl Roth GmbH & Co. KG, Germany) and 66 µl STC. Subsequently, the required volume of protoplasts in STC were added and the final volume was set to 600 µl with STC. After mixing by flicking the tube, samples were incubated for 10 min at RT before 3.75 ml of 60% PEG 4000 was subsequently added. After thorough mixing by inversion, samples were incubated for 20 min at RT. Afterwards, STC was added to a final volume of 15 ml, reducing the viscosity of the mixture prior to centrifugation. Protoplasts were pelleted for 10 min at 3200×g^− 1^ and 20 °C using a swing-out rotor centrifuge. The supernatant was discarded and the pellet was resuspended in 2 ml of regeneration broth (2% MEB and 500 mM sucrose, Carl Roth). After incubation without agitation for 24 h at 25 °C, 200 µl of the mixture was plated on osmotically stabilized malt-extract agar (500 mM sucrose, 34 g/L MEA) containing 100 µg/ml nourseothricin. Plates where incubated at 25 °C and visible mycelial growth occurred after 10 to 14 days.

### Genomic DNA isolation, PCR, and Southern blot analyses

For routine gDNA extraction from isolated using the E.Z.N.A. HP Plant & Fungal DNA Kit (Omega Bio-tek) or DNeasy Plant Mini Kit. For generation of biomass from putative transformants, MEA supplemented with 100 µg/ml nourseothricin was used and a nylon filter membrane (Carl Roth) was placed on top of the agar to avoid collection of agar medium during mycelium harvest. Progenitor control gDNA was extracted similarly but with nourseothricin omitted from MEA. For Southern blotting DNA was extracted based on our previously published protocol for *A. niger* [30]. In brief, biomass was snap-frozen in liquid nitrogen, grinded in a pestle and mortar, following a phenol-chloroform DNA extraction step, including degradation of RNA by RNAse treatment. PCR was conducted using PhireGreen Polymerase (ThermoFisher Scientific) and Southern blots probing the *tef1* promoter conducted using our established protocols (Supplemental Table 1 [30]).

### RNA isolation and qRT-PCR

RNA from 10 days old mycelium grown on MEA with 100 µg/ml nourseothricin was added into TRI Reagent (Zymo Research) containing glass beads (1–2 mm) and disrupted for 60 s at 5 m/s using a Mini‑Bead‑Mill‑Homogenisator (VWR). Total RNA was isolated using the Direct-zol RNA Miniprep Kit (Zymo Research) skipping the on-column DNA digestion. DNA was digested with DNA-free DNA Removal Kit (ThermoFisher Scientific), and 500 ng of total RNA were used for cDNA synthesis using random hexameric primers and the RevertAid H-Minus FirstStrand cDNA Synthesis Kit (ThermoFisher Scientific). The cDNA was diluted 10-fold and 2 µl per 10 µl reaction volume were used with the Blue SyGreen 2x qPCR Mix (Biozym) in a 2-step PCR-protocol combining primer binding and target amplification at an annealing temperature 60 °C for 15 s on an Aria Mx Real-Time PCR System (Agilent).

### Fluorescent microscopy

Putative transformants of *F. fomentarius* were cultivated on MEA  supplemented with 100 µg/ml nourseothricin for 10 days, with progenitor control grown on MEA. Afterwards, squares (approx. 10 mm x 10 mm) were cut with a scalpel and placed on water agar (2% agar-agar in water). After 7 days, cubes from the water agar overgrown with a monolayer of mycelium were used for brightfield and fluorescence microscopy. A DM5000B-CS (Leica) equipped with a K5-14403617 camera (Leica) at a magnification of 400x was used with an exposure time of 1000 ms for mCherry and 500 ms for eGFP.

### DNase activity assay

*F. fomentarius* was cultivated in liquid CM in shake flasks in 4 days at 25 °C, *A. niger* in liquid CM in shake flasks in 3 days at 30 °C. Biomass was harvested by filtering through Miracloth and washed 5 times with 20 ml sterile water. Supernatants were immediately used in the DNase activity assays. Filtered biomass was immediately snap frozen and ground in liquid nitrogen before resuspending ~ 0.1 g wet weight in 10 ml sterile water to obtain crude cell lysate solutions. 8 µl of either supernatant or crude cell lysate solution was incubated with 2 µl of ~ 500 ng/µL PCR or plasmid product for the indicated time. EDTA, MgCl_2_ or ZnCl_2_ were added to the final concentration indicated. For boiled controls, supernatant or cell lysate solutions were incubated at 100 °C in a heat block for 15 min, before being cooled to RT before use. Following the indicated incubation time, DNA was purified using innuPREP DOUBLEpure Kit (Innuscreen, Berlin) and analysed using gel electrophoresis.

## Results

### Transformation of *F. fomentarius*

**Fig. 1:**
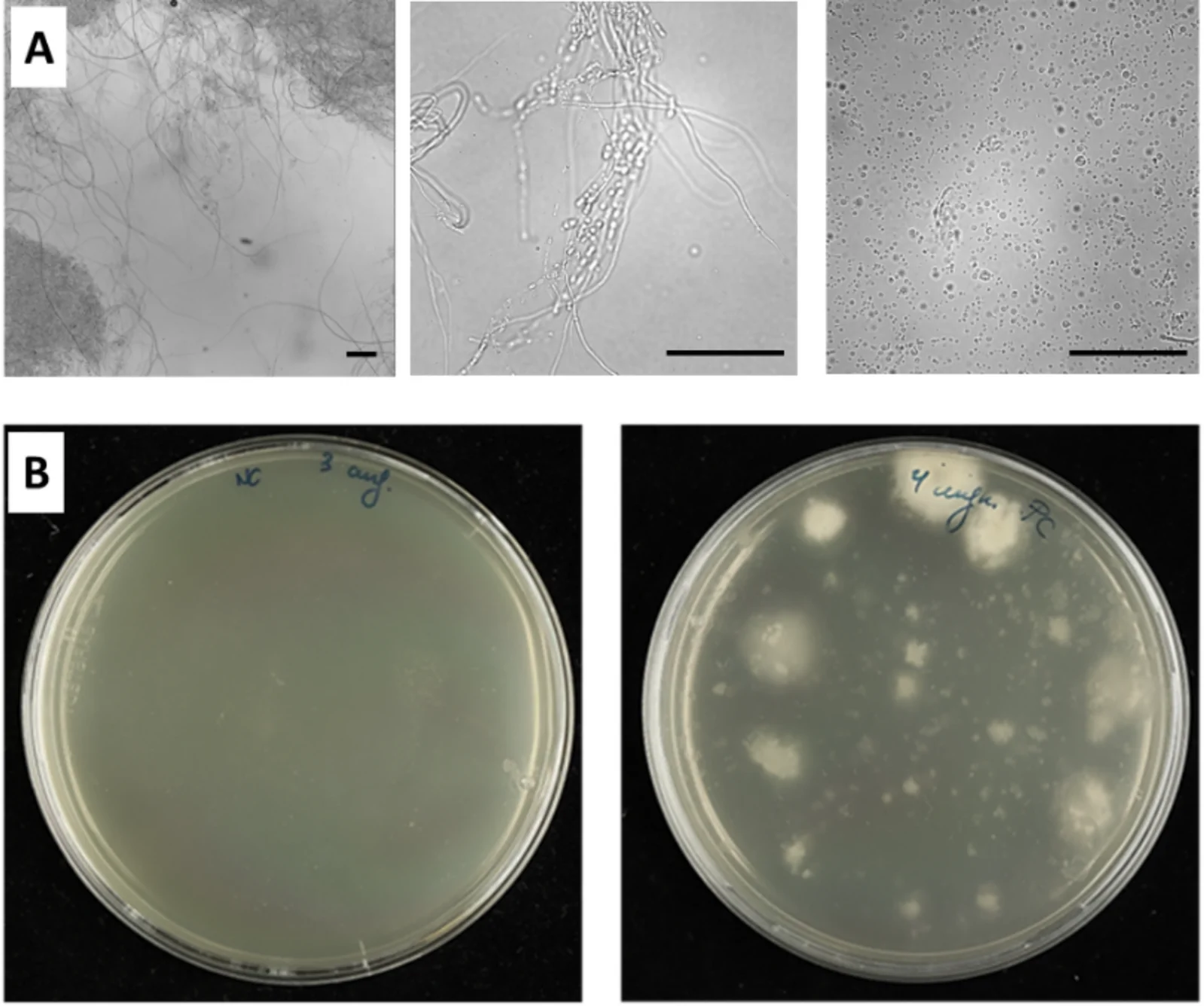
Protoplast-mediated transformation of *F. fomentarius* using nourseothricin selection. **A** Representative images of *F. fomentarius* biomass and purified protoplasts. Starting material used for protoplasting from either static or shaken cultivation consists of compact biomass with long hyphae extending into the liquid media (left panel). Hyphae were visibly degraded in protoplast solution (75 min incubation, middle panel). Purified *F. fomentarius* protoplasts prior to PEG-mediated transformation (right panel). Note small amounts of contaminating hyphal fragments and cellular debris. Scale bar is 100 μm. **B** Representative images of primary transformation plates following 10 days incubation. Negative control (left panel) consists of protoplasts incubated with sterile water and resulted in very low frequency of background colonies. Transformation with pHom2 (right panel) resulted in 10–30 putative transformants per plate. Note high variation in colony size and morphology of the putative transformants.

Two main genetic transformation methods exist for basidiomycetes, including protoplast- and Agrobacterium-mediated transformation [31]. Preliminary protoplastating experiments demonstrated that *F. fomentarius* mycelium from both static and shaken liquid cultivation generated abundant protoplasts (Fig. 1A) using our protocols developed for the ascomycete *Aspergillus niger* with only minor modifications (see Methods). A crucial aspect for efficient protoplast-mediated transformation is starting biomass, and we favoured 5-day old static liquid cultivation in Fernbach flasks with a large surface area to volume ratio over shaken flasks as preliminary observations suggested better growth and less mycelial debris in purified protoplasts (Supplemental File 2 and data not shown). Our preliminary experiments also suggested that the multi-enzyme blend VinoTaste^®^ Pro generates more viable protoplasts for *F. fomentarius* compared to an enzyme mix obtained from *Trichoderma harzianum* or the enzyme Yatalase (Supplemental Table 2). We thus established the transformation protocol based on protoplasting *F. fomentarius* with the cell wall lysing enzyme blend VinoTaste^®^ Pro (see Methods).

Transformation of agaricomycetes can be achieved using the protein synthesis inhibitor nourseothricin as selection and nourseothricin N-acetyl transferase encoding gene (*natR*) as resistance marker [20]. We thus generated preliminary evidence for functionality of this marker system in *F. fomentarius* by transformation of protoplasts with plasmid pHom2 (kindly provided by Prof. Han Wösten [20]). It encodes a *natR* sequence under control of a *S. commune gpdA* promoter. Protoplast-based transformation achieved > 1 CFU/µg pHom2 from six technically replicated experiments (data not shown). This equates to approximately 10–30 putative transformants per agar plate (Fig. 1B). Negative control plates (derived from protoplasts incubated with sterile water instead of pHom2) resulted in absence or very low numbers of CFUs (e.g., 0–3 CFUs, Fig. 1B). Based on these data we hypothesized that nourseothricin and *natR* could be used in further transformation attempts for *F. fomentarius*.

Native promoters are important for efficient expression of a desired gene in agaricomycetes [32]. We therefore reasoned that for future genetic engineering projects, the *natR* cassette should be placed under control of a native *F. fomentarius* promoter. Translation elongation factor (*tef*) promoters are well established in fungi for both high-level and constitutive expression, and we thus conducted in silico analyses of the promoter region of *F. fomentarius tef1* (gene ID 1241408). This revealed a predicted intron, initiator element, downstream promoter element, 1 GC-box, 3 CAAT boxes and a transcription start site (Supplemental File 3), which supported the use of this sequence as a promoter. Interrogation of our previously published transcriptional datasets spanning biomaterial, laboratory growth, and *in planta* cultivation conditions (*n* = 27 [33]), confirmed *tef1* was consistently highly expressed, ranking on average 5th most highly expressed out of 14,754 genes (with a lowest ranking of 18th out of 14,754 predicted genes).

**Fig. 2:**
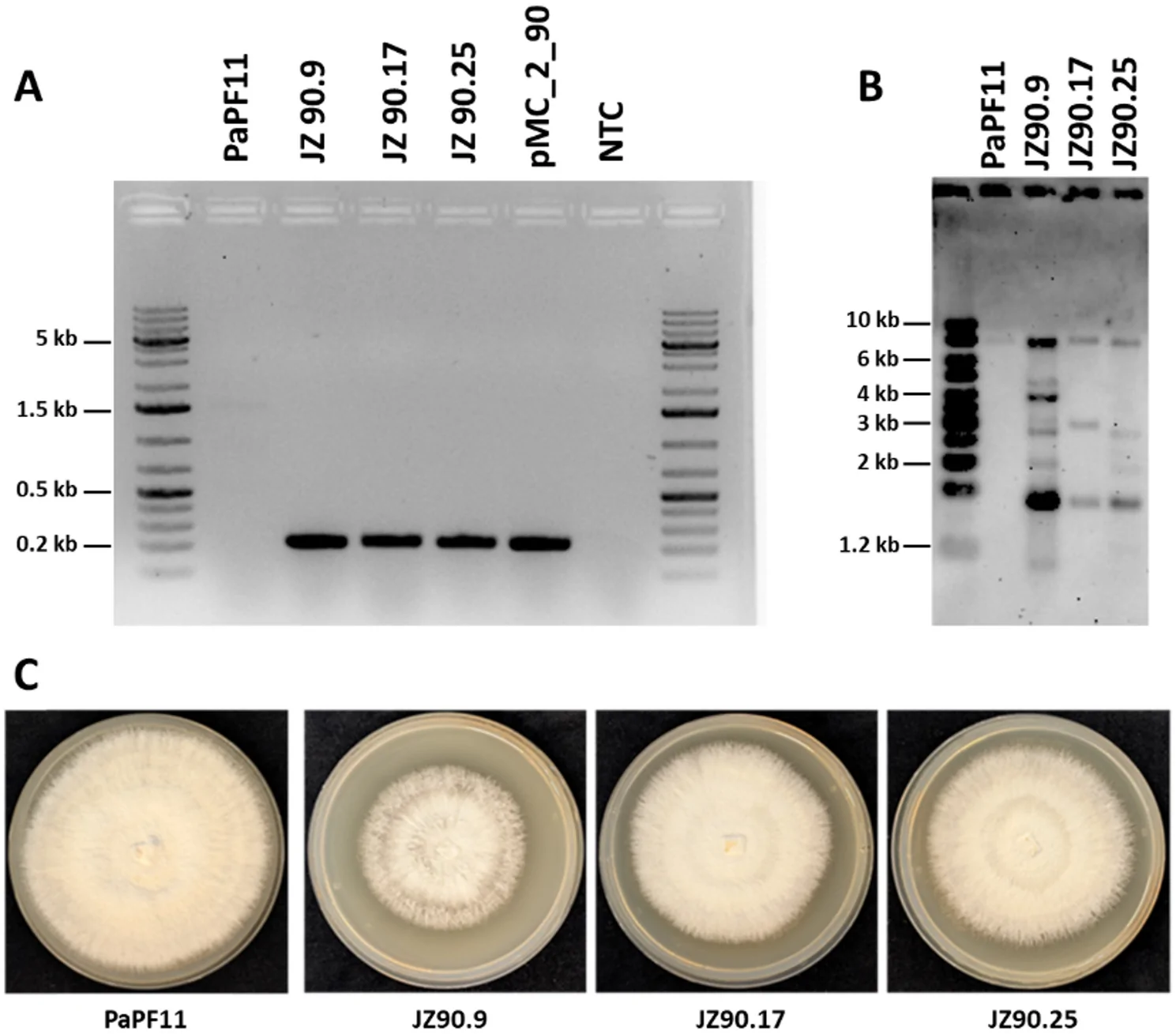
Molecular confirmation of *F. fomentarius* transformation and radial growth analysis of mutant strains. **A** Exemplar image confirming the *natR* cassette is exclusively observed in PCR using genomic DNA extracted from transformant isolates. Primers JZ 72 and JZ 73 amplify a 216 bp region of the *natR* cassette. This amplicon was present in all transformant derived PCRs, pMC_2_90 positive control, yet absent when progenitor PaPF11 DNA was used as template. NTC is a no template control PCR which was routinely analysed alongside all diagnostics. **B** Southern blot analysis confirms multicopy integration of the *tef1* promoter in transformant isolates. Genomic DNA was digested with KpnI and probed for a portion of the *tef1* promoter. Wild-type isolate PaPF11 displayed a single copy at the expected size (~ 8 kb), which was present in all mutant strains. Additionally, transformants demonstrated clear multicopy integration of the *tef1* promoter encoded in the pMC_2_90 derived transformation cassette. **C** Representative images of PaPF11 control and mutant isolate colony growth on solid agar. Growth assays were conducted in triplicate, representative pictures are shown.

We therefore used our established modular cloning protocol [34] to generate plasmid pMC_2_90, whereby *natR* is under control the *F. fomentarius tef1* promoter. Additionally, this plasmid encoded 5’ and 3’ 250 bp targeting regions for deletion of a predicted *ku70* encoding gene (gene ID 1195489). Generating *ku70* deletion mutants has been a highly successful strategy improve homologous recombination- and thus integration of exogenous DNA cassettes at desired loci- through inhibiting non-homologous end joining [35]. A linear transformation cassette was generated by PCR using pMC_2_90 as template, and this was transformed into protoplasts alongside a pre-assembled CRISPR/Cas12a ribonucleoprotein (RNP) complex targeting the *ku70* locus (see Methods). Putative transformants were subcultured on nourseothricin, after which the presence of the *natR* cassette was confirmed by PCR (Fig. 2A). However, *ku70* deletion was unsuccessful as determined by diagnostic PCR (Supplemental File 4). In order to provide evidence for stable integration of the transformation cassettes into the *F. fomentarius* genome, DNA was extracted from three PCR-confirmed transformants and the *tef1* promoter region probed using Southern blot (Fig. 2B). This assay confirmed multicopy integration of the transformation cassette into each transformant genome.

Next, we analysed the three transformants confirmed via Southern blot using radial growth assays on complete agar. This revealed minor but detectable impacts on colony morphology for isolates JZ90.17 and JZ90.25 (Fig. 2C). In contrast, mutant JZ90.9 demonstrated marked reduction in radial growth and altered colony morphology (Fig. 2C). Interestingly, this mutant also demonstrated the most frequent marker integration events in Southern blot analyses (Fig. 2B), which may indicate a metabolic cost of expressing numerous *natR* copies, or, alternatively, integration of one or more cassette(s) at loci necessary for normal *F. fomentarius* growth or metabolism.

Taken together, we conclude that stable integration of exogenous DNA cassettes in *F. fomentarius* can be mediated via protoplast-based transformation. Additionally, our data demonstrate that expression of a *tef1::natR* cassette does not cause severely defective growth and colony formation in all engineered strains. Nevertheless, mutant JZ90.9 strongly suggests that transformants may display phenotypes which are dependent on cassette copy number or integration locus.

### Fluorescent protein markers for *F. fomentarius*

**Fig. 3:**
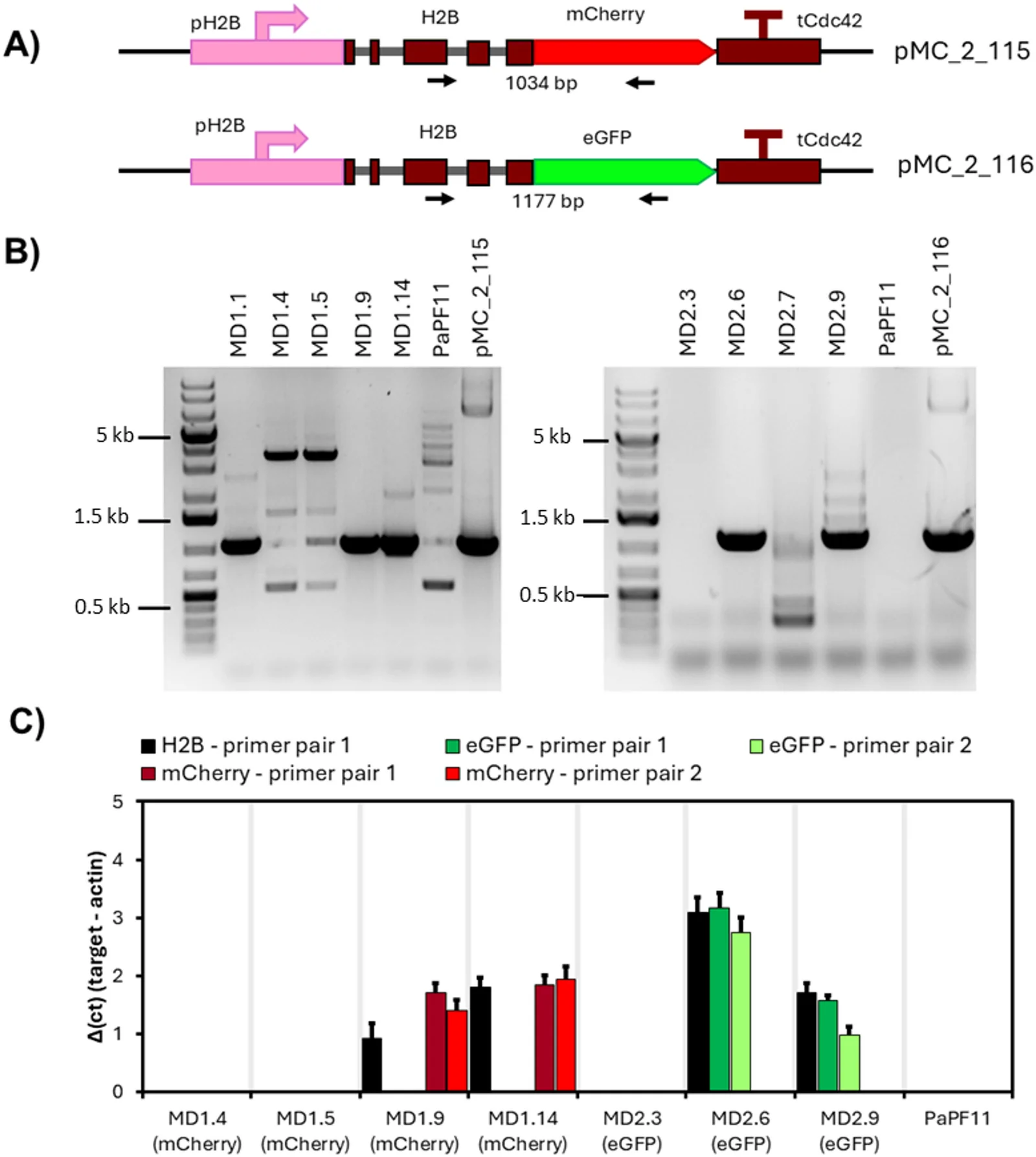
*F. fomentarius* is capable of expressing fusion proteins. **A** Schematic representation of the fusion protein constructs on pMC_2_115 and pMC_2_116 used for transformation of *F. fomentarius* PaPF11. **B** PCR on genomic DNA extracted from selected clones confirms the presence of expression cassettes for H2B-eGFP and H2B-mCherry only in a subset of clones. Primer binding sites and amplicon sizes are depicted in (**A**). **C** Expression of H2B and respective fused fluorescent protein in *F. fomentarius*. Values are represented as ∆(ct) relative to the expression of endogenous actin (gene ID 1265750) as measured by qRT-PCR. For H2B, a single primer pair was used while for mCherry and eGFP, 2 primer pairs were used.

Fluorescent markers are crucial to study the position, function, and turnover of a gene product. In order to deliver these tools for *F. fomentarius*, we generated pMC_2_115 and pMC_2_116, which respectively express mCherry or eGFP. In both instances, fluorescent protein encoding genes were under control of a *S. commune* H2B promoter and nuclear localisation sequence (Fig. 3A) with *F. fomentarius* selection mediated via *tef1*::*natR*. Transformation was conducted as described above, with PCR used to confirm integration of *natR* in putative mutants (Fig. 3B). We initially screened functionality of the H2B promoter for driving expression of H2B localisation signal, mCherry or eGFP in *F. fomentarius* using qRT-PCR (Fig. 3C). This assay demonstrated that several transformants which encode the *natR* cassette did not express either H2B or respective fluorescent gene (e.g., MD1.4, MD1.5, MD2.3 Fig. 3C). We suggest this is either due to integration of a non-functional truncated cassette, or recombination of exogenous DNA into transcriptionally inactive chromosomal loci. Encouragingly, expression of the H2B and either eGFP or mCherry encoding genes was detected in other transformants at moderate to high levels, i.e., 10–30% of actin transcript level (Fig. 3C). For eGFP expressing mutants MD2.6 and MD2.9, we observed distinct expression levels which is consistent with non-targeted multicopy integration observed for other transformants (Fig. 2B).

**Fig. 4:**
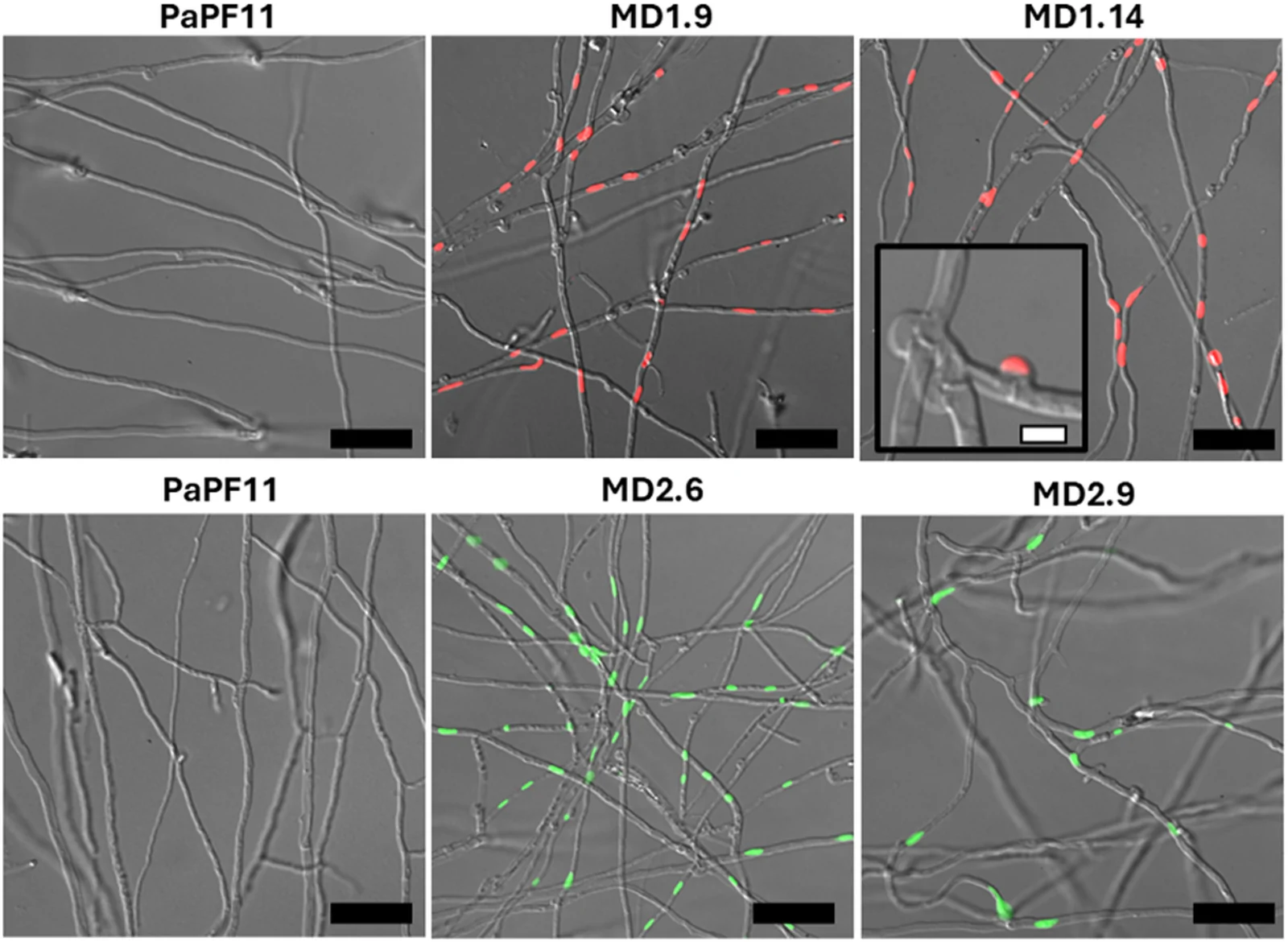
Fluorescence microscopy confirms expression of H2B-eGFP and H2B-mCherry fusion proteins in *F. fomentarius*. Two colonies transformed with plasmid pMC_2_115 (MD1.9 and MD1.14) or pMC_2_116 (MD2.6 and MD2.9) where chosen. Fluoresce indicates localization of the fusion protein to the nucleus. The black scale bar represents 25 μm length, the black inset picture for MD1.14 shows a nucleus inside a clamp connection, the white scale bar represents 5 µm.

Mutants passing PCR and qRT-PCR screening were analysed using fluorescent microscopy, which confirmed functionality of both mCherry and eGFP in transformant hyphae (Fig. 4). Autofluorescence from progenitor control was minimal, and in both instances, fluorescence was detected in punctate locations consistent with the nucleus. Taken together, we conclude that both fluorescent proteins are fully functional in *F. fomentarius* and that the *S. commune* H2B promoter is appropriate for heterologous gene expression. Additionally, we hypothesize that the H2B localisation signal likely directs proteins to the nucleus of *F. fomentarius* as shown previously for other fungi [27].

### *F. fomentarius* displays DNase activity which could impact transformation

An unexpected observation during this study was the routine failure of standard genomic DNA extractions from progenitor and transformant strains as well as fluctuating numbers in primary transformants. We reasoned this was due to high levels of *F. fomentarius* DNase activity. In order to test this hypothesis, we incubated PCR amplicons or plasmids with *F. fomentarius* crude cell lysate before analysis of DNA integrity using gel electrophoresis.

**Fig. 5:**
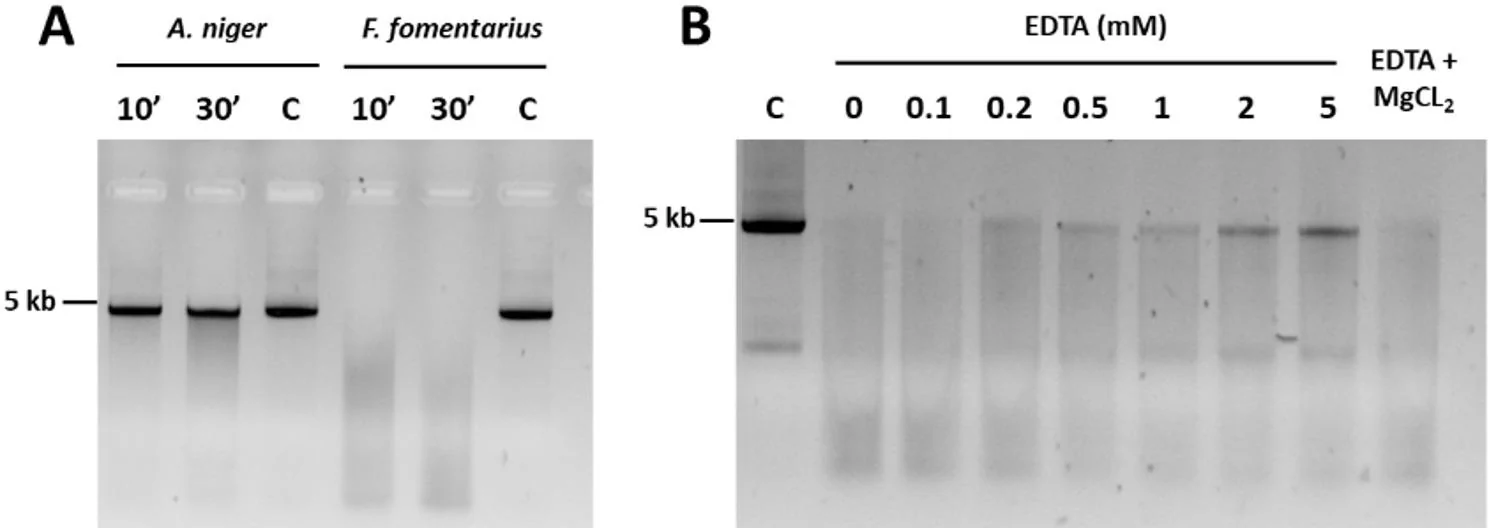
*F. fomentarius* DNase activity. **A** Crude cell lysate from either *F. fomentarius* or *A. niger* control was incubated with 1 µg of linear PCR product (size 4.6 kb) at room temperature for the indicated time period. Control lanes denoted with ‘C’ consisted of boiled cell lysate followed by incubation with PCR product for 30 min. *F. fomentarius* samples fully degraded DNA within 10 min, which was not observed in the boiled control or *A. niger* samples. **B** EDTA can inhibit *F. fomentarius* DNase activity, supposedly via chelation of MgCl_2_ [36]. Control lane denoted with ‘C’ consisted of the untreated PCR product. *F. fomentarius* crude cell lysates were incubated with PCR product for 10 min at room temperature and the indicated concentration of EDTA. We observed reduced DNase activity with increasing EDTA concentration. Degradation of PCR product at 5 mM EDTA could be achieved by supplementing 5 mM MgCl_2_ into the incubation mixture (final gel lane).

Remarkably, 1 µg of linear PCR product was fully degraded by cell lysate solutions of *F. fomentarius* within 10 min at room temperature (Fig. 5A). We also observed minor, but clearly detectable DNase activity in *F. fomentarius* culture supernatants when incubated for 60 min (Supplemental File 5). DNase activity was barely detectable using supernatant or lysate from the model industrial cell factory *A. niger* in this assay (Fig. 5A and Supplemental File 5). Such endonuclease activity was also confirmed in *F. fomentarius* crude cell lysate by rapid degradation of plasmid DNA (Supplemental File 6).

Interestingly, DNase activity was moderately inhibited by 5 mM EDTA (Fig. 5B), but the inhibition became counteracted by the addition of Mg²⁺, i.e. DNase activity became fully restored. This strongly suggests that the observed DNase activity is dependent on divalent cations with Mg²⁺ as a potential catalytic cofactor. In contrast, ZnCl₂ also inhibited DNase activity, but this inhibition was not reversed by Mg²⁺ supplementation (Supplemental Fig. 7), suggesting that Zn²⁺ inhibits the DNase activity through direct metal substitution or binding at the catalytic site rather than by simply depleting Mg²⁺.

Taken together, we consider DNase activity to be a surprising technical challenge both in terms of genomic DNA extraction and possibly for genetic engineering of *F. fomentarius*. Although beyond the scope of this proof of principle study, we are currently working to reduce DNase activity during transformation (e.g., via EDTA). We hypothesize exogenous DNA cassettes may be degraded during transformation, and possibly after cassettes have entered the *F. fomentarius* cell.

## Discussion

Innovation in materials science is vital to meet UN sustainability goals [12, 37]. To maximize green applications of fungal-based materials, numerous groups have successfully established state-of-the-art genetic engineering systems for wood-decaying basidiomycetes [15–20]. In this study, we have generated proof of principle that *F. fomentarius* can be genetically transformed.

Various technical challenges have been overcome in this work, firstly with regards to proof that the *natR* marker and nourseothricin selection is functional in *F. fomentarius*. Use of pHom2, and three derivative plasmids constructed in this study, generated abundant putative clones on primary transformation plates with minimal growth on negative controls (Fig. 1B). Thus, we conclude that similar to many agaricomycetes, *natR* and nourseothricin can be used as an excellent dominant selection system for *F. fomentarius*. Obviously, expanding the suite of markers - such as carboxin, hygromycin, phleomycin, or auxotrophy - is an urgent priority.

Next, we have confirmed at the molecular level that *F. fomentarius tef1* and *S. commune* H2B promoters are suitable for expression of *natR* and two heterologous fluorescent protein encoding genes respectively (Fig. 2–4). Validation of two promoters opens the exiting possibility for future gain-of-function studies in *F. fomentarius*, whereby a transformation marker utilizes one promoter (e.g., *tef1*), and a gene of interest is constitutively expressed by another (e.g., H2B). It should be noted that we assume a *S. commune gpdA* promoter (which drives expression of *natR* in pHom2) is also functional based on recovery of nourseothricin resistant clones (Fig. 1), although this was not confirmed via PCR or qRT-PCR in this study.

Additional technical advances reported in this work are the validation of mCherry and eGFP, which we speculate will enable analyses of protein subcellular localisation, turnover, or even fungal-substrate interactions. Indeed, the H2B nuclear localisation sequence localized fluorescent proteins to punctate areas consistent with the nucleus. We therefore suggest this targeting is functional in *F. fomentarius*. More generally, expression of two fluorescent proteins provides additional confirmation that genetic transformation and expression of exogenous cassettes have been achieved in this study.

Significant technical challenges remain, however. Firstly, our preliminary attempts to disrupt the *ku70* locus via genome editing and homologous flanks were unsuccessful. Clearly, precise targeting - whether using classical homology-based protocols and/or CRISPR-Cas genome editing - is a prerequisite for gene functional analyses via knock-out or disruption in this fungus. Also, overexpression studies are technically easier and more accurate with regards to identifying phenotype-gene function relationships if cassettes are targeted to a specific locus [38, 39]. We will thus optimize targeting efforts in *F. fomentarius* through future engineering studies. Secondly, our Southern blot analyses confirmed multicopy integration of exogenous cassettes in the recipient genome. This is a common yet additional limitation to genetic engineering of non-model fungi, as random, multiple integration can impact cassette expression levels or cause deletion/disruption of important regulatory loci or genes.

Finally, an unexpected discovery was the level of DNase activity of *F. fomentarius* crude cell lysate and detectable activity in culture supernatants. Whilst not prohibitive for transformation of this fungus (Fig. 2), we speculate that DNase activity could be reducing transformation efficiency, and, possibly, targeting of the *ku70* cassette during disruption efforts. This is based on the observation that both linear and plasmid DNA is rapidly degraded (i.e., 1 µg in < 10 min for a PCR product), which we speculate may impact abundance and integrity of cassettes which reach the *F. fomentarius* nucleus. Although beyond the scope of this study, we are currently working to reduce DNase activity in this fungus (e.g., magnesium chelation via EDTA). We suggest that similar DNase activity could be reducing transformation efficiency in other material forming fungi.

## Conclusions

We provide conclusive proof *F. fomentarius* can be genetically transformed using the tools and approaches developed in this study. This toolkit will enable over-expression of a desired gene of interest for functional analyses. However, off-target, multicopy cassette integration events must be carefully considered when conducting and interpreting such experiments. Given that eGFP and mCherry were functional in *F. fomentarius*, we consider these reporters high priority tools for future subcellular localization studies. We have also identified DNase activity as a potential challenge for genetic engineering, and speculate that this may limit targeted approaches, including gene deletion or disruption. Future studies from our group will further develop engineering of this important biomaterial-producing fungus.

## Supplementary Information

Below is the link to the electronic supplementary material.

Supplementary Material 1.

Supplementary Material 2.

## Data Availability

All data generated or analysed during this study are included in this published article and its supplementary information files.
