# Supplementary material for: Genetic transformation and fluorescent protein expression in the biomaterial-producing fungus Fomes fomentarius
Source: Fungal Biol Biotechnol. 2026 Nov 2;13:20. doi: 10.1186/s40694-026-00228-w (PMC13726337; doi:10.1186/s40694-026-00228-w)
Supplement: Supplementary file 1 [file 40694_2026_228_MOESM2_ESM.docx]

Supplemental Table 1. Primers used in this study

| **Primer** | **Sequence** | **Template** |
| --- | --- | --- |
| 35_Gibson_2_91_rev | GCCGCATCAGACGCGCCGAGAAATTCTCTGAGCGCCTCCTTTTGTACC | Amplification of backbone of pMC_2_91 for Gibson assembly |
| 36_Gibson_2_91_fw | TGGCATTGAGTTTCCTGGTGCGGAGCGCCGGGGTTCC |  |
| 37_Gibson_p40p14_fw | GGTACAAAAGGAGGCGCTCAGAGAATTTCTCGGCGCGTCTGATGCGGC | Amplification of fusion protein cassettes from p14 and p40 |
| 38_Gibson_p40p14_rev | GATGGAACCCCGGCGCTCCGCACCAGGAAACTCAATGCCA |  |
| 3585_Ffom_act_1265750_fw-1 | TTAACCCCAAAGCCAACCGT | qPCR of *F. Fomentarius* actin |
| 3586_Ffom_act_1265750_rv-1 | AGGCATAGAGGGAGAGCACA |  |
| 45_ScomH2B_qPCR_1_fw | AAGAAGACCCGCAAGGAGAC | qPCR of S. commune H2B |
| 46_ScomH2B_qPCR_1_rev | CTTCTTCGAGTACGCCGCAA |  |
| 49_eGFP_qPCR_1_fw | CGACCACTACCAGCAGAACA | qPCR on eGFP primer pair 1 |
| 50_eGFP_qPCR_1_rev | CGCTTCTCGTTGGGGTCTTT |  |
| 51_eGFP_qPCR_2_fw | AGGACGACGGCAACTACAAG | qPCR on eGFP primer pair 2 |
| 52_eGFP_qPCR_2_rev | TTCTGCTTGTCGGCCATGAT |  |
| 53_mCherry_qPCR_1_fw | GGACGGCGAGTTCATCTACA | qPCR on mCHerry primer pair 1 |
| 54_mCherry_qPCR_1_rev | CAGCTTCAGCCTCTGCTTGA |  |
| 55_mCherry_qPCR_2_fw | CGGCGAGTTCATCTACAAGGT | qPCR on mCHerry primer pair 2 |
| 56_mCherry_qPCR_2_rev | TCTGCTTGATCTCGCCCTTC |  |
| 28_gpd_natR_FW | CGGAAGACAAAATGAGTGTCAGTCCCGTCCTTCTCTCC | Verification of *natR* presence |
| 21_natR_PCR_rev_1 | GCAGGGCATGCTCATGTAGA |  |
| 45_ScomH2B_qPCR_1_fw | AAGAAGACCCGCAAGGAGAC | Verification of mCherry presence |
| 54_mCherry_qPCR_1_rev | CAGCTTCAGCCTCTGCTTGA |  |
| 45_ScomH2B_qPCR_1_fw | AAGAAGACCCGCAAGGAGAC | Verification of eGFP presence |
| 50_eGFP_qPCR_1_rev | CGCTTCTCGTTGGGGTCTTT |  |
| 3515_nat_pICH41308_fw_1 | CGGAAGACAAAATGGCCACCCTCGACGACACGGCTTACC | Cloning of *natR* into MoClo entry vector pICH41308, removal of BpiI site |
| 3516_nat_pICH41308_rev_1 | CGGAAGACAACACGGTGTCGGTGGTGAAGGACCCATC |  |
| 3517_nat_pICH41308_fw_2 | CGGAAGACAACGTGTTCCGCGTCACCGCCACCG | Cloning of *natR* into MoClo entry vector pICH41308, removal of BpiI site |
| 3518_nat_pICH41308_rev_2 | CGGAAGACAAAAGCTCAGGGGCAGGGCATGCTCATGTAG |  |
| 3975_tScom-SC3-fw | CGGAAGACAAGCTTGATCGATCCTGTGAGTGCACGTCAG | Amplification of *S. commune* SC3 terminator from phom2 for cloning into MoClo entry vector pICH41276 |
| 3976_tScom-SC3-rev | CGGAAGACAAAGCGCCTCCTTTTGTACCCCCTTACTTCG |  |
| JZ_6_pFftef_long_fw1 | CGGAAGACAAGGAGTGGACGAGCGGTGCTCGG | Amplification of *F. fomentarius tef1* promoter cloning into MoClo entry vector pICH41295 |
| JZ_8_pFftef_rev1 | CGGAAGACAATTCTCGAAAATTTCAGATGATCGATAGAGATCTTGGC |  |
| JZ_9_pFftef_short_fw2 | CGGAAGACAAAGAACGTCCAAGGGTACATAAACCTTAAGACC |  |
| JZ_10_pFftef_short_rev2 | CGGAAGACAACATTTTTTGATATAGATCTAATATCGTCGAAAGTCAGTGCACTGTG |  |
| ku70_5pHR250_fw | GTGGTCTCAGGAGCGTGACTTGCTGAACAAGACGG | Cloning of 250-bp 5’-UTR of Ffomku70 into MoClo vector pICH47732 |
| ku70_5pHR_universal_rev | GTGGTCTCAAGCGGGGTCAACTTCAACAGTGTTTCTATGG |  |
| ku70_3pHRuni_fw | GTGGTCTCAGGAGCGCCGGGGTTCCATCTAATCC | Cloning of 250-bp 5’-UTR of Ffomku70 into MoClo vector pICH477/” |
| ku70_3pHR250_rev | GTGGTCTCAAGCGCCTTGAGTTCATCCGACGCT |  |
| Lvl-0-screening-FW | AATAGGCGTATCACGAGGC | universal Level 0 MoClo vector screening primers |
| Lvl-0-screening-R | AGTCAGTGAGCGAGGAAGC |  |
| Lvl-1-screening-FW | CACACTGCGACGACTTTTGCATAATCAGTC | universal Level 0 MoClo vector screening primers |
| Lvl-1-screening-R | CCGCCAAATGTTTGAACGAATTG |  |
| pT7_FnCpf1_fw | ATGTAATACGACTCACTATAGGTAATTTCTACTGTTGTAGAT | T7 promoter for annealing with sgRNA primer to generate transcript template for T7 polymerase |
| ff_sgRNA_1A_snpcorr | TCTCAGAGGAAAGTCAGTTAATCTACAACAGTAGAAATTA | sgRNA 1 targeting *ku70* in *F. fomentarius* |
| ff_sgRNA_1B_snpcorr | TCTACTTTCTCTTCCTGTAAATCTACAACAGTAGAAATTA | sgRNA 2 targeting *ku70* in *F. fomentarius* |
| ff_ku70_locus_f2 | CTGGTCTGGAGGCATGATGT | Testing *ku70* locus integrity |
| ff_ku70_locus_r2 | CTTCAACTGGTCGACACGGA | Testing *ku70* locus integrity |
| ff_cPCR_natR_2_r | CGCGACCCTATGACATCGAA | Testing *ku70* locus integrity |
| ff_tef_probe_f | GTGCTCGGAGGGTAAAGAGA | Amplify portion of *tef1* promoter for Digoxigenin labelling and Southern blot probing |
| ff_tef_probe_rev | GGCGACAAGACAGTCATGAAA | Amplify portion of *tef1* promoter for Digoxigenin labelling and Southern blot probing |

Supplemental Table 2: Overview of enzyme formulations tested for protoplasting Fomes fomentarius PaPF11. Released protoplasts per g mycelium wet weight after the final washing step are reported. The cultivation method for mycelium, the used enzyme formulation and amount as well as incubation time and temperature are given. Preliminary data from single experiments are shown. nd = not determined.

| **Enzyme formulation** | **Concentration [mg/g mycelium wet weight]** | **Temperature [°C]** | **Incubation time [h]** | **Cultivation of *Fomes fomentarius*** | **Released protoplasts per g mycelial wet weight**  **[n x 10^6^]** |
| --- | --- | --- | --- | --- | --- |
| Yatalase (Takara) | 100 | 30 | 4 | shaken | 0.22 |
| Lysing Enzyme from *Trichoderma harzianum* (Sigma Aldrich) | 25 | 37 | 5 | shaken | 0.12 |
|  | 25 |  | 4 | Shaken | 0.45 |
|  | 25 |  | 3 | Shaken | 0.4 |
|  | 50 |  | 3 | Shaken | 1.18 |
|  | 50 |  | 3.5 | Shaken | 6.81 |
| VinoTaste Pro (Novozymes) | 100 | 30 | 5 | Shaken | nd |
|  | 250 |  | 5 | Shaken | 0.16 |
|  | 250 | 37 | 2.75 | Shaken | 1.65 |
|  | 250 |  | 2.5 | Shaken | 8.91 |
|  | 250 |  | 1 | Shaken | 4.15 |
|  | 250 |  | 3 | Static | 14.1 |
|  | 250 |  | 1 | Static | 5.92 |
